# Selections

**Published:** 1871-06

**Authors:** 


### Selections.

### *On the Therapeutic Value of Diaphoresis.* By DR. W. LEUBE, Lecturer and First Assistant to the Medical Clinic, Erlangen.

[*Deutsches Archiv f. klin. Med.*, vii, 1, p. 24, 1870.]

These remarks are appended to a longer article intended to prove, by a series of very careful clinical and chemical investigations, a reciprocal bearing or antagonism between the cutaneous and the renal secretions. Hence the author confines himself, in discussing the therapeutical effects of diaphoresis, to renal affections chiefly.

If we accept as a fact the point developed by the author's investigations: that the secretions of the kidney and of the skin bear a formal reciprocal relation to one another, the therapeutic action of diaphoresis in cases of disturbed or suppressed function of the kidneys becomes of far greater importance than we could hitherto ascribe to it; its symptomatic indication grows an *indicatio morbi;* it becomes a therapeutic measure whose efficacy corresponds equally to practical and physiological experience. Among the remedies recommended against a disease, that one deserves preference which has been proved statistically, by large series of results from unbiased observations, to be most efficacious, whether its mode of action is explained or not; in the latter case, our failure to understand the action is owing to deficient knowledge of the pathological process or deficient physiological reasoning. But when, as in the case of Bright's disease, a number of alleged remedies are proposed in competition, we must always, if we wish to abstain from sentimental therapeutics, give preference to the one that is backed by physiological experiment. In this respect, diaphoresis holds the first place among the remedies recommended against diseases of the kidneys.

Although he does not doubt the usefulness of diaphoresis in all renal affections, the author speaks chiefly of chronic Bright's disease, because it is the most frequent, and the one concerning which the greatest number of accurately observed cases are recorded. Complete cure of well determined chronic Bright's disease is not brought about by diaphoresis, any more than by any other remedy; it is well known to be an extremely rare exception. We must not expect too much from any remedy; no one hopes for a restoration of destroyed parts, or full functional recovery of the lungs in chronic pneumonia; we content ourselves with relative functional capacity. In the kidney, the remedy is intended to remove the albuminuria; the kidney, which after the cessation of the inflammatory process, is contracted, but may still contain enough working tissue to avert the dangers of suppression of the urinary excretion, should be free from congestion and phenomena of abnormal conditions of blood-pressure. According to the beautiful experiments of Stokvis, the existence of a hæmatogenetic albuminuria is highly improbable, whilst the nephrogenetic albuminuria can be shown to be due solely to lesions of circulation in the kidney, and not to alterations of its epithelium or other tissue elements. The cases observed in this clinic, as well as those reported by Liebermeister, prove that diaphoresis is capable of achieving complete success in a number of cases,—or at least that degree of success which is possible under the special conditions of the case.

Much more evident is the utility of energetic diaphoresis in *uræmia.* So long as it had not been demonstrated that the skin is capable of excreting a considerable amount of nitrogenized products, the dangers of so severe a remedy were, rightly enough, anxiously weighed in this serious disease; the more so as until lately the derivatives of urea were considered as the true poison in this condition. Physiological experience now compels us to deny those substances all importance, and to recognize as the cause of uræmic symptoms the retention or deficient excretion of all the effete matters of the body, among which it is likely, (according to Voit), that potash plays an especially prominent part. But whether we adhere to the older theory, or the new: ammonia, urea and potash can all be withdrawn from the blood by means of powerful diaphoresis; the mechanical conception, also, of the nature of uræmia, based on the hypothesis of œdema of the brain, offers no *a priori* objection to the diaphoretic method, as the latter could only favor the absorption of the dangerous œdema. Hence the author believes that, in view of the unreliable character of all the remedies recommended against this affection which is considered to be incurable, an immediate *energetic* diaphoresis is the first and only measure indicated, even though a negative result in this pernicious disease would seem thus far to oppose this train of reasoning.

Difficult to explain, but the more surely established by experience, is the purely systematic *hydragogue*, and in some cases of dropsy permanent, *effect of diaphoresis.* Refraining from hypotheses on the subject, the author recommends a *practical method of employing diaphoresis in dropsy*, which is far less exhausting than the usual total "packing," and yields no less gratifying results. Packing the body in woolen blankets after a hot bath is in many cases of high-graded dropsy, especially in heart disease, impossible, certainly very irksome, and possibly dangerous by reason of exhaustion even to fainting; the author has therefore employed diaphoresis *locally.* He places, for instance, the lower extremity in a bath of 98 deg. F., increasing the temperature to 108 deg., and after allowing it to remain in the hot bath for half or three-quarters of an hour, wraps it in india-rubber cloth, in case of very sensitive skin interposing a piece of linen.

Over the rubber cloth is rolled a thick flannel bandage, and perhaps a small woolen blanket; and, if the patient will bear it, the extremity thus enveloped may be subjected to a gentle kneading. In this manner the patients lie for four to six hours, sometimes the whole night, without discomfort; sometimes only they would complain of itching, especially when the india-rubber was in direct contact with the skin. When this process is repeated and continued daily without interruption, the absorption of lighter grades of œdema can be accomplished in a few days; and even in cases of very considerable dropsy, the author effected a diminution of the circumference of the leg by ten centimetres in four days. Although this local diaphoresis has not as decisive an effect as the general method, it can be recommended for its simplicity, as well as for the fact that the temperature of the limb which is to be made to perspire can be elevated higher than would be possible, without danger and discomfort, in a total bath.

Finally, the author recommends, on the strength of a large experience, that the bath be given in the morning; perspiration is more easily effected in the morning than after noon. This point has been determined by observations of pulse and respiration, before and during the packing, the figures always being considerably higher in the afternoon or evening, when the sweating also gives more discomfort to the patient, than in the morning.—*St. Louis Medical and Surgical Journal.*

---

*Bromide of Potassium in Croup.* By S. B. Kieffer, M.D., Carlisle, Pa.

The peculiar and acknowledged action of the bromides would indicate that they have a special control over the various nervous affections of the larynx, trachea, and other organs of the throat; but their special use in membranous croup has hitherto not been well established.

If, in this short paper, it shall appear that in this disease also, which has always been to the profession the occasion of much concern, there is a special disposition to yield to their power, I trust the profession will give the subject such attention as will demonstrate fully its truth. More than four years ago, my friend Dr. W. W. Dale was called, in my absence, to a case which he regarded as one of genuine membranous croup, and for which he prescribed an emetic, followed by repeated doses of calomel. On the following morning we saw it together, and the correctness of his diagnosis seemed very evident; but the little patient, a year old, had found no relief. Under the peculiar constitutional habits of the child, we both regarded the case as hopeless, and expected a rapid decline. For reasons which I shall explain hereafter, and as a matter of experiment, I gave the following prescription:

| | |
|---|---|
| ℞ Brom. of Potassium, - - - - - - - - | gr. xx; |
| Chlorate of Potassa, - - - - - - - - - | gr. x; |
| Ipecac, - - - - - - - - - - - - - | gr. j; |
| Ext of Liquorice. . - - - - - - - - - | ʒss; |
| Water, - - - - - - - - - - - - | f℥ijss. |

M. S.—A teaspoonful every hour, with directions to report after eight hours.

After the fourth dose the child became easier, the breathing less difficult and dry, and after the fifth hour it fell into a quiet and comfortable sleep, lasting three hours, when we saw it, and, to our mutual surprise, found it greatly relieved and much better. The treatment was continued, and at the end of the third day the patient was discharged. Since that time, both Dr. Dale and myself have depended exclusively upon this treatment, more or less modified according to circumstances, and *uniformly with success.* So confident am I that, when timely and judiciously administered, it has the power of arresting both the inflammation and the deposit of false membrane, that I now approach my patients, thus suffering, without fear, and with little anxiety. As there is no nausea, and the disease yields without emesis, it had not occurred to me to have demonstrative proof of actual membrane formed, until several months ago, when I was called into the country to see a patient aged four years, and who had now been suffering distressingly to the fifth day. This case, it seemed to me, was unmistakably one of membranous croup, and had been treated regularly by emetics, mild cathartics, blisters, etc., but it had stubbornly resisted them all, and was steadily growing worse, while the symptoms were apparently of the most aggravated character. My prognosis at this stage was decidedly unfavorable; but I gave the patient the bromide, in mixture, as before indicated, and on the following morning found it apparently much better. I now gave it, by means of the steam atomizer, a solution of bromide of potassium and chlorate of potassa topically, and continued the medicine, as before, every hour. On the following morning I found marked improvement, and ordered the medicine to be continued; and now, on again using the atomizer, assisted by my friend Dr. E. A. Grove, violent coughing ensued, and piece after piece of disintegrated false membrane was thrown off, demonstrating beyond a doubt its actual presence. The medicine was continued, and at the end of the fourth day the patient was regarded as out of danger, and on the following day was discharged.

I could not, after experiments from good authority, regard the bromide of potassium as *a solvent*, so to speak, of false membrane; nor have I ever thus regarded it; but I do believe, and on this principle I have prescribed it, that just in proportion as it is a *sedative* to the cerebro-spinal system directly, so it is a stimulant, indirectly, to the nerve-filaments and circulation of the throat; and, as the inflammation in membranous croup is usually, if not always, of the asthenic character, it has the power by its specific action of equalizing the circulation and arresting the fibro-albuminous deposit. And when the disease is not too severe, or has not progressed too far, the system thus, by its own inherent power, will be equal to the task of repairing the evil.

The experience of my friend W. W. Dale, M.D., who has been using the same treatment, though in the earlier stages of the disease he carries the use of ipecac to nausea, and frequently combines quinine with the mixture (a necessity rarely called for, I think, when the nausea is avoided), is substantially the same; and I here speak by his authority when I state that membranous croup, spasmodic croup, and laryngitis, alike, have lost for him, as they have also for me, nearly all that dread and anxiety with which he once met them.

I trust my professional brethren will give it a trial.—*Medical Times.*

---

*Therapeutical Value of Chloral.* Extract from a paper read at a meeting of Liverpool Chemists' Association, held December 22, 1870.

If we review the pages of the medical journals for the therapeutical effects of hydrate of chloral, we shall find many cases where its action has been attended with marvellous results. There does seem not a little danger of its being erected into a kind of panacea for all the ills that flesh is heir to—of its true worth and fame suffering from too indiscriminate use, and from the administration of some of the impure compounds which are being supplied. Its value, however, is too real for actual collapse by its abuse; but its repute may be, and doubtless has been, dangerously compromised.

We find it employed in cases of "maniacal paroxysms," "delirium tremens," "traumatic tetanus," chorea, diarrhœa, whooping cough, convulsions (epileptic or otherwise), with more or less benefit; it allays vomiting, and prevents sea-sickness; in puerperal mania it is well reported of; in fact, as a sleep-compeller it is, in a very large number of cases, unrivaled; for while in power opium alone can be compared with it, there is this superiority to opium, that its use entails no unpleasant after symptoms, no head-ache, no nausea, no anorexia, no constipation, whilst the sleep it produces is gentle, calm and continued; at least, this is the general rule, but, of course, there are exceptions, and medical men complain that its administration is attended with uncertain results, and that its quality is not so good as it was when first introduced; but even with true hydrate of chloral we must expect to find exceptional cases, so long as human beings differ so greatly in temperament, constitution, and sensibility to the action of medicine.

That hydrate of chloral ought to be perfectly pure when used in medicine is unquestionable; the substitution of alcoholate is quite sufficient to produce most of the ill effects attributed to chloral. In fact, instead of being a hypnotic, it has a tendency to produce mental excitement, as ordinary stimulants.

The dose of hydrate of chloral is from 5 grains to 30 or 40 grains, according to the purpose for which it is required. A case is on record where 100 grains were taken accidentally without any evil results; but I am informed that there is danger in continued small doses. Very unexpected results have, in a few instances, occurred. And here I would strongly caution pharmaceutists not to prescribe its use themselves, or supply it to the public without the sanction of a medical man.

Hydrate of chloral has been successfully administered as an antidote to strychnia.

Hydrate of chloral cannot, in consequence of its chemical properties, be administered in the shape of pills or in the form of powder; it is, therefore, necessary almost to confine its use to solutions. For dispensing purposes, Liebreich recommends a solution of the hydrate in its own weight of water. In small doses it can be given without the addition of a corrective, but simply dissolved in distilled water.

There are several pharmaceutical preparations in which the hydrate of chloral is disguised, or its taste modified, in various ways. Of the syrups, containing 10 grains of Liebreich's hydrate in each dram, one made with syrup. pruni virg. is used in America; it is most palatable. Another is made with syr. tolu; others with syr. flor. aurant., syrup. cort. aurant. (as suggested by Liebreich). Another is flavored with almonds (Ferris). There is also a draught containing half dram chloral, with syrup tolu, tinct. ginger and peppermint water. Lozenges containing 1 grain hydrate of chloral in each, are manufactured by Messrs. Meggeson & Co.

Spiritus chloralis is made by Savory & Moore. It has a very agreeable taste and smell, but I was not able to obtain any deposit upon evaporating a little.

Limousin's capsules are known to contain alcoholate of chloral, because true hydrate cannot be secured in a gelatinous envelope.

In describing and dispensing hydrate of chloral, it should be borne in mind that no corrective with alkaline reaction can be employed with it, because such an administration would bring about the transformation of the substance.

In concluding this paper, I must add that I have no interest whatever in putting forward the claims of Liebreich's manufacture, further than a feeling of moral duty to the medical profession, pharmacists and the public, together with the *conviction* that other manufactures which have come under my notice do not attain the desired standard. It appears that the importers of this article now know a guaranteed hydrate of chloral and an unguaranteed hydrate of chloral. There is a guarantee to the consumer, which is the protection of the hydrate manufactured under Liebreich's supervision: this is a registered trade mark. It is offered in three forms—cake, crystal and powder; but the action of the cake is more to be relied upon. Each product should be kept in well-stoppered bottles. The large quantity which the bottles with the registered trade mark contain is, I think, a drawback to its more universal application; and I think, if the agents of this manufacture could be induced to supply it in smaller bottles,—say from 1 oz. upwards,—with the registered label on each bottle, and could produce it at a cost more in proportion with the competition, they would not only further the objects of the discoverer by more satisfactory and uniform results being produced, but also benefit mankind in general.—*Pharm. Journ., Lond., Jan.* 7, 1871.—*Am. Jour. Pharmacy.*

---

***Treatment of Intermittent Fever.***

Dr. S. C. Lacey, Laceyville, Pa., sends us the following plan of treatment of intermittent fever:

℞. Gelseminum, Fld. Ext., - - - - - } āā ℥ i.

Liquor Potass. Arsenit. - - - - - }

Dose—20 drops three times per day, letting the patient drink freely of hop tea during the interval, or not, as you see fit.

I have treated a large number of cases both East and West, and never knew a patient to have more than one chill after commencing the medicine. I continue the medicine for one week, then I omit for five days, and then give two days, and then omit five days again. Do that way for four weeks and then discontinue the medicine.

I think there is nothing like gelseminum in the treatment of bilious remittent fever, also in infantile remittent. In uterine hemorrhage it is a valuable remedy. I have also found it efficacious at the commencement of labor and when the pains are inefficient, combined with fluid ext. ergot.—*Jour. Mat. Med.*

